# Burden of Disease Assessment of Ambient Air Pollution and Premature Mortality in Urban Areas: The Role of Socioeconomic Status and Transportation

**DOI:** 10.3390/ijerph17041166

**Published:** 2020-02-12

**Authors:** Soheil Sohrabi, Joe Zietsman, Haneen Khreis

**Affiliations:** 1 Zachry Department of Civil Engineering, Texas A&M University, College Station, TX 77840, USA; sohrabi.s@tamu.edu; 2 Center for Advancing Research in Transportation Emissions, Energy, and Health (CARTEEH), Texas A&M Transportation Institute (TTI), College Station, TX 77843, USA; zietsman@tamu.edu; 3 Barcelona Institute for Global Health (ISGlobal), Centre for Research in Environmental Epidemiology (CREAL), 08003 Barcelona, Spain

**Keywords:** burden of disease, air pollution, premature deaths, attributable deaths, road traffic, socioeconomic inequities, United States

## Abstract

With recent rapid urbanization, sustainable development is required to prevent health risks associated with adverse environmental exposures from the unsustainable development of cities. Ambient air pollution is the greatest environmental risk factor for human health and is responsible for considerable levels of mortality worldwide. Burden of disease assessment (BoD) of air pollution in and across cities, and how these estimates vary according to socioeconomic status and exposure to road traffic, can help city planners and health practitioners to mitigate adverse exposures and promote public health. In this study, we quantified the health impacts of air pollution exposure (PM_2.5_ and NO_2_) at the census tract level in Houston, Texas, employing a standard BoD assessment framework to estimate the premature deaths (adults 30 to 78 years old) attributable to PM_2.5_ and NO_2_. We found that 631 (95% CI: 366–809) premature deaths were attributable to PM_2.5_ in Houston, and 159 (95% CI: 0-609) were attributable to NO_2_, in 2010. Complying with the World Health Organization air quality guidelines (annual mean: 10 μg/m^3^ for PM_2.5_) and the US National Ambient Air Quality standard (annual mean: 12 μg/m^3^ for PM_2.5_) could save 82 (95% CI: 42–95) and 8 (95% CI: 6–10) lives in Houston, respectively. PM_2.5_ was responsible for 7.3% of all-cause premature deaths in Houston, in 2010, which is higher than the death rate associated with diabetes mellites, Alzheimer’s disease, or motor vehicle crashes in the US. Households with lower income had a higher risk of adverse exposure and attributable premature deaths. We also showed a positive relationship between health impacts attributable to air pollution and road traffic passing through census tracts, which was more prominent for NO_2_.

## 1. Introduction

About 55% of the world’s population lived in urban areas in 2018, and this percentage is expected to increase to 68% by 2050 [1]. To ensure that the benefits of urbanization are equally distributed and that the risks are mitigated, urban areas need to be sustainably developed to meet population needs such as housing, transportation, health care, and other infrastructures [2]. While sustainable development of cities refers to meeting the needs of the present without compromising the ability of future generations to meet their own needs [3], unsustainable development of cities are associated with health risks for the present and future generation, including those from air pollution, climate change, unsafe drinking water, among others [2,4]. Despite the fact that urban air quality is a part of the assessment of sustainability of cities [4,5], according to a World Health Organization’s (WHO) study on more than 4300 cities worldwide, 80% of the urban population lives in urban areas which do not comply with the WHO air quality guideline value for Particulate Matter with a diameter equal or less than 2.5 micrometers (PM_2.5_) alone [6]. It has been shown that exposure to air pollution is associated with numerous health issues such as lung cancer [7], respiratory diseases [8] including chronic obstructive pulmonary disease [9], asthma [10], cardiovascular diseases [11,12], and stroke [9], to name a few. Conservative estimates from the WHO attributed 4.2 million annual deaths worldwide to ambient air pollution in 2016 [13]. Other studies showed that land transport-related air pollution, in particular, is responsible for one-fifth of deaths from air pollution in the United Kingdom, the United States (US), and Germany [14].

While burden of disease (BoD) assessments of air pollution at national, regional, and global levels are useful, city governments, who are more agile, can only act within their jurisdictions and mitigate adverse exposures and impacts at the city level. In this context, we quantify and analyze the BoD attributable to air pollution, in the form of premature deaths, focusing on a large urban area suffering from urban sprawl and witnessing rapid population growth. We analyze the spatial variation of estimated premature deaths across the city to investigate the possible role of exposure to road traffic. The unequal distribution in health impacts of air pollution in an urban area is examined in terms of population socioeconomics.

Mortality attributable to air pollution in urban areas was quantified in a number of studies [15,16,17,18,19,20]. One study in Bradford, UK, explored and showed that the exposure to air pollution and its attributable burden of mortality inversely correlated with population socioeconomic status proxied by household income and with ethnic minorities [19]. However, no study has formally investigated whether living in areas with a higher level of road traffic correlates with the spatial variation of air pollution health outcomes. Such an analysis can be used to demonstrate the significance of road traffic in the BoD estimates when running full-chain BoD assessment models is time-consuming and cost-intensive [21].

From a methodological standpoint, previous BoD assessment studies share a similar methodology for quantifying the health burden which could be attributable to air pollution. Generally, the baseline exposure level is obtained and compared with either level of exposure recommended by health authorities or a no-exposure scenario (i.e., elimination of the exposure). Then, the relative risk of the detrimental health outcome is calculated for each exposure difference using exposure-response functions (ERF) extracted from the literature. Finally, the attributable health outcome for the population is estimated for each exposure difference using the baseline mortality rates and population data [20]. The air pollution BoD analyses have been conducted at different spatial levels including census-tract level [20,22], neighborhoods [23], lower super output area [19], and city-wide estimations [24]. The spatial level of the analysis is often dependent on the availability of data. For example, Lelieveld et al. captured the contribution of air pollution to mortality for a 100 × 100 km spatial resolution [14]. Air pollution data are estimated using air quality models [15,18,19,20,25]. Different studies used different pollutants including PM_2.5_ [19,20,23], Nitrogen dioxide (NO_2_) [19,26], and Ozone [14], where higher BoD was attributed to PM_2.5_ and NO_2_ compared to Ozone. Air pollution BoD assessment studies in cities are mainly conducted in Europe (e.g., [15,18,19,20,26]). In the US, an analysis of the health impacts of air pollution by Goodkind et al. showed that PM_2.5_ was responsible for 107,000 deaths in 2011 [27]. Kheirbek et al. in 2016 estimated the BoD of traffic-related PM_2.5_ in New York City and examined the relation between the estimated BoD and poverty at the neighborhood level [23].

In our analysis, we ran a BoD assessment for two criteria air pollutants: NO_2_ and PM_2.5_, among others. NO_2_ and PM_2.5_ are traffic-related pollutants which have been associated with stronger adverse health effects including premature mortality. NO_2_ is, however, considered more specific to traffic-related air pollution [28]. Air pollution concentrations were estimated by a land-use regression (LUR) model and a universal kriging framework. We quantified premature deaths which could be attributable to these exposures using standard BoD assessment methodology, separately for each pollutant. The finer the spatial resolution of the analysis, the better the insight one can gain into health inequality issues, contributors, and high-risk spots which can be effectively targeted by policies. We, therefore, ran our analysis at the census tract level to capture spatial variations at a fine scale. To examine the health inequalities, we compared the distribution of air pollution attributable health impacts by household median income across the city. We also compared the BoD attributable to air pollution when complying with the WHO air pollutant guidelines versus the US National Abient Air Quality Standard (NAAQS) by the US Environmental Protection Agency (EPA). As a case study, we focused on the city of Houston, Texas; the fourth most populated city in the US and a rapidly growing urban area with no zoning regulations. The methodology of this study is applicable to other cities, and the results can benefit both city planners and health professionals to detect high-risk spots in cities and plan interventions accordingly. Additionally, the results can raise public awareness of the health impacts associated with air pollution and its spatial distribution across cities and promote dialogue with policymakers and other stakeholders.

## 2. Materials and Methods

### 2.1. Study Setting and Definitions

The BoD attributable to air pollution was quantified in the city of Houston for the year 2010, the year for which we had air pollution models. The city of Houston is the largest city in Texas with 636.5 square miles (1646 square km) land area and 2,099,451 residents in 2010 [29]. The city is located in three US counties; Harris, Fort Bend, and Montgomery. The BoD assessment was conducted at the finest reasonable spatial resolution: the census tract level. The rationale behind assessing the BoD at the census tract level is twofold. First, the mortality data was only available at the county level, and so approximations were required to assign baseline mortality rates to a finer spatial level. To minimize the error of these approximations, and yet investigate the spatial distribution of health outcomes, we chose to limit the spatial resolution of this study to the census tract level. Second, analyzing the road traffic spatial variations (discussed in subsequent sections) in finer resolutaion can give a better understanding of the relation between health impacts of air pollution and road traffic. Consequently, 592 census tracts were included in this study, which were fully or partially located within Houston city’s boundaries.

We quantified the health impacts in the form of attributable premature deaths. Premature death is defined as a measure of unfulfilled life expectancy [30], which is considered as the number of deaths before reaching the expected age of death in a population. The life expectancy in the US was 78.7 years old in 2010 [31]. The risks of mortality in association with NO_2_ and PM_2.5_ were sourced from meta-analysis studies for individuals older than 30 years old (details are provided in subsequent sections). Hereafter, the term premature death refers to the death of individuals aged 30 to 78 years old who died in a natural way (as opposed to accidents and suicides).

### 2.2. Input Data

The data used in this study were collected from multiple sources—namely, the US census bureau, Centers for Disease Control and Prevention, and Texas Department of Transportation, as described in the following sections.

#### 2.2.1. Population, Socioeconomic, and Geographic Data

Population and socioeconomic data were collected from the US Census Bureau for 2010 at the census tract level along with the census tracts’ geographic characteristics. We stratified the estimated BoD by household economy using the median household income at the census tract level, as sourced from the US Census Bureau. The average households’ median income in the city of Houston, in 2010, was 52,857 dollars. City of Houston geographical limits was sourced from the city’s open data portal which was used to identify the census tracts within the city’s boundaries (retrieved from https://cohgis-mycity.opendata.arcgis.com/datasets/houston-city-limit).

#### 2.2.2. Mortality Data

The baseline mortality data for Texas in the year 2010 was sourced from the Centers for Disease Control and Prevention (CDC) (https://wonder.cdc.gov/mcd.html). The mortality data was available both in the form of the number of deaths and crude mortality rates (Crude mortality rate is the total number of deaths of residents in a county divided by the total population for the county (for a calendar year) and multiplied by 100,000) at the county level with 95% confidence intervals (CI). To quantify premature deaths attributable to air pollution, the number of all-cause natural deaths for people aged 30–78 years old was used in this study. Given that the city of Houston is located in three counties: Harris, Fort Bend, and Montgomery, the mortality data for these three counties was collected. We distributed the number of mortality cases (available at the county level) across census tracts proportionally based on their population size. In 2010, a total number of 8667 all-cause premature deaths (natural deaths excluding accidental mortalities) were reported in the city of Houston (30–78 years old). The summary statistics of the mortality data at the census tract level are reported in Table 1.

#### 2.2.3. Air Pollution Data

**Figure 1:**
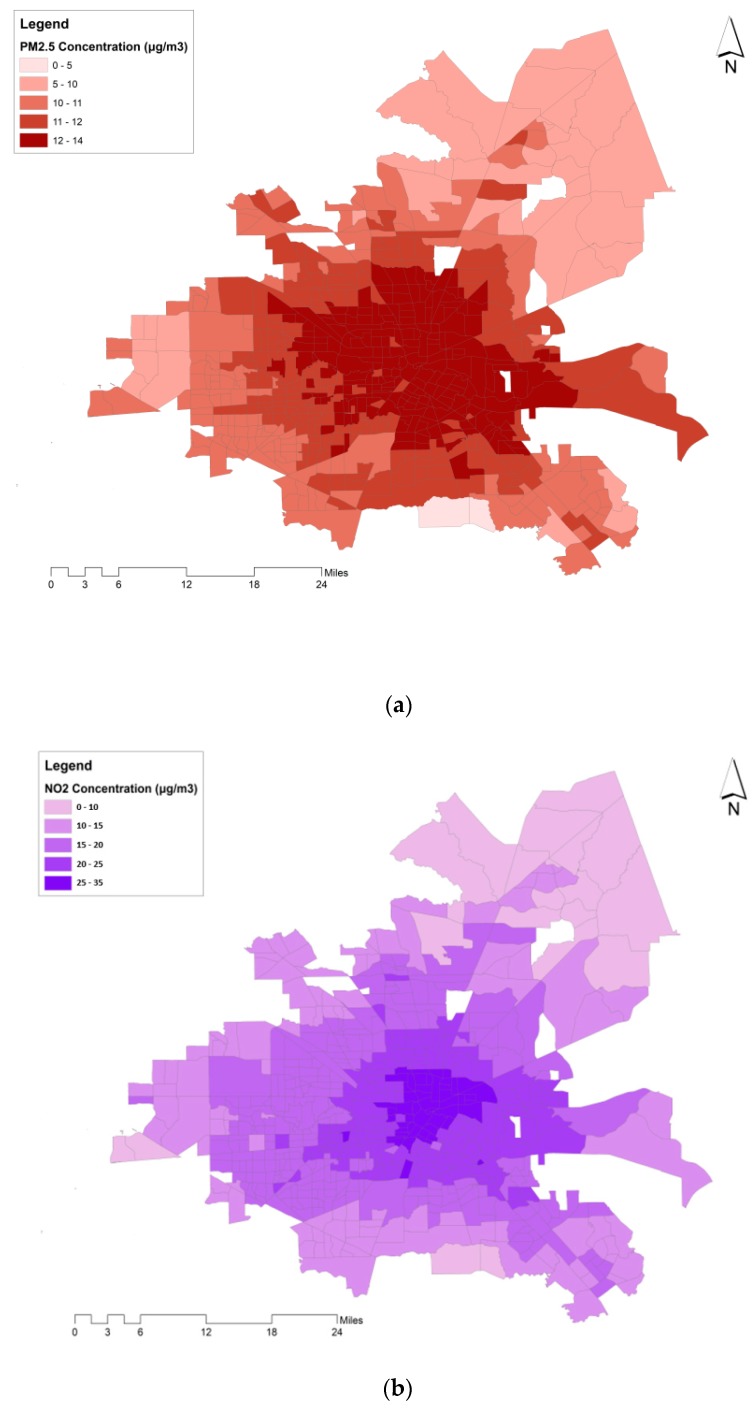
(**a**) PM_2.5_ and (**b**) NO_2_ annual average concentrations across the city of Houston at the census tract level, in 2010.

NO_2_ and PM_2.5_ concentrations were sourced from a previously published and validated LUR model and a universal kriging framework, also employed in our previous study [32]. We used the annual average concentrations in μg/m^3^ for the year 2010 at the centroid of each census tract. The data was originally estimated at the centroid of the census blocks which are one level smaller than census tracts. We assigned the area-weighted average of concentrations at census blocks (that are contained within each census tract) to census tracts. NO_2_ concentrations were converted from ppb to μg/m^3^ through multiplying by 1.88 [33]. Table 1 provides a detailed summary of pollutant concentrations across the city. The spatial distribution of air pollutants is also shown in Figure 1. In the following, we briefly discuss the models used for estimating NO_2_ and PM_2.5_ concentrations.

The NO_2_ estimates were obtained from a LUR model, developed by Bechle et al. [34]. In brief, the model uses satellite data and EPA air quality monitor readings of NO_2_ concentrations, alongside several covariates (for example, impervious surfaces, elevation, major roads, residential roads, and distance to the coast) to estimate NO_2_ concentrations. The final model we used has a relatively high predictive power at unmeasured locations which was tested using a hold-out cross-validation with good model performance (R^2^ = 0.82); which is comparable with other continental-scale NO_2_ LUR models [35,36,37].

On the other hand, annual average concentrations of PM_2.5_ were estimated using data from 17 years (1999–2015). The data were derived from regulatory monitors and estimates were constructed in a universal kriging framework [38]. Partial least squares were estimated for model performance from hundreds of geographic variables, including land use, population, and satellite-derived estimates of land use and air pollution. Hold-out cross-validation (CV) indicated good model performance (10-fold CV-R^2^ = 0.85). Annual PM_2.5_ concentrations were estimated at the census tract centroids (with similar procedure as described above).

#### 2.2.4. Road Traffic

In this study, the annual averaged daily vehicle mile traveled per area (VMTA) was used to investigate the relation between road traffic and the air pollution health burden, at the census tract level. VMTA represents the density of vehicle mile traveled (VMT) at a census tract. The vehicle mile traveled (VMT) was calculated by aggregating the multiplication of the road segment length and annual daily traffic (ADT) passing through all road segments located within a census tract. Then, the VMT was divided by the census tract’s area. Equation (1) shows the VMTA calculation for each census tract:

(1) $$\mathrm{VMTA}\;=\;\frac{\mathrm{ADT}.\mathrm{Road}\;\mathrm{Length}}{\mathrm{Census}\;\mathrm{Tract}\;\mathrm{Area}}\;\left(\frac{\mathrm{veh}.\mathrm{mi}}{{\mathrm{mi}}^{2}}\right)$$

In addition to the roads located within the census tract, the possible impacts of roads near the census tract’s boundary were taken into account. According to the WHO, PM_2.5_, and NO_2_ concentrations decrease to background concentrations within 100–150 m (328–492 feet) of a roadway [39]. To this end, we included the VMT passing through all roads within 492 feet distance of the census tract’s boundary to estimate the VMTA of that census tract.

We identified the roads located within a 492 feet distance of the census tract boundary using ArcGIS and included those in VMTA calculations. Road network and ADT data were sourced from the Texas roadway inventory data by the Texas Department of Transportation (https://www.txdot.gov/inside-txdot/division/transportation-planning/roadway-inventory.html). The data was not available for 2010, and therefore, the ADT data from 2011 was used. We assumed that the spatial distribution of road traffic across census tracts is consistent between 2010 and 2011. A summary of road traffic statistics is presented in Table 1. 

### 2.3. Burden of Disease Assessment Model

We used a standard BoD assessment framework previously developed in the literature [20]. In brief, the inputs to the BoD model included the NO_2_ and PM_2.5_ concentrations, as well as the baseline all-cause death rate in the studied region. Next, the Relative Risk (${\mathrm{RR}}_{\mathrm{diff}}$) of all-cause mortality in association with the difference between current concentrations and the counterfactual concentrations were estimated. Equation (2) presents the ${\mathrm{RR}}_{\mathrm{diff}}$ calculations for a linear ERF.

(2) $${\mathrm{RR}}_{\mathrm{diff}}=\mathrm{RR}\times \frac{\left(E_{\mathrm{current}}-E_{\mathrm{counterfactual}\;\mathrm{exposure}}\right)}{{\mathrm{RR}}_{\mathrm{unit}}}$$

where RR is the relative risk as extracted from the literature, $E_{\mathrm{current}}$ represents the current concentration level, $E_{\mathrm{counterfactual}\;\mathrm{exposure}}$ represents the counterfactual exposure level, and ${\mathrm{RR}}_{\mathrm{unit}}$ is the exposure unit of RR obtained from the original ERFs. Then, the population attributable fraction (PAF) was calculated using Equation (3). The PAF represents the ratio of premature deaths attributable to air pollutants from all-cause deaths for the difference between current exposure levels and the counterfactual exposure levels.

(3) $$\mathrm{PAF}=\frac{{\mathrm{RR}}_{\mathrm{diff}}-1}{{\mathrm{RR}}_{\mathrm{diff}}}$$

**Figure 2:**
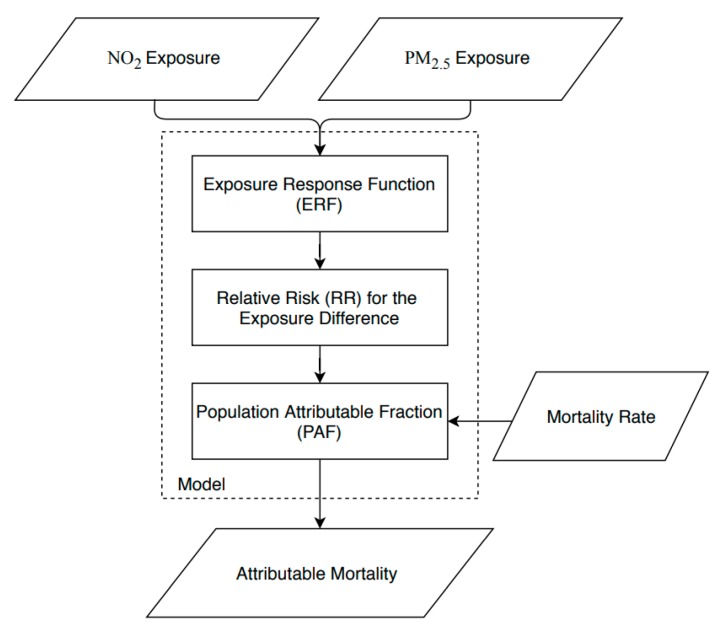
Burden of disease assessment framework.

Finally, the attributable deaths were estimated using the mortality rate and population counts for people aged 30–78 years old, and the estimated PAF (using Equation (4)). The employed BoD assessment framework is presented in Figure 2. This procedure was used for estimating the attributable premature deaths across the 592 census tracts, separately for each pollutant.

(4) Attributabl Mortality = PAF×Mortality rate ×Population counts

### 2.4. Exposure-Response Functions

We extracted the ERF for NO_2_ and PM_2.5_ from two meta-analyses. The first meta-analysis included data from 22 cohort studies with a total of 367,251 participants and was used for the ERF of NO_2_ and mortality [40]. Based on the documented ERF which associated natural deaths with NO_2_, the RR of deaths per 10 μg/m^3^ NO_2_ was estimated as 1.02 (95% CI: 0.99–1.04) for individuals older than 30 years. This RR was adjusted for sex, calendar time, smoking status, smoking intensity, smoking duration, environmental tobacco smoke, fruit intake, vegetable intake, alcohol consumption, body-mass index (BMI), educational level, occupational class, employment status, marital status, and area-level socioeconomic status [40]. Note that the lower limit of the RR: 0.99 implies that no adverse health effect is associated with 10 μg/m^3^ increase in NO_2_ exposure.

The ERF for PM_2.5_ was extracted from a meta-analysis by WHO [41]. This meta-analysis was performed on 14 studies and resulted in RR for different regions. For the US, using a linear ERF, the overall RR of natural deaths associated with PM_2.5_ was estimated as 1.07 (95% CI: 1.02–1.12) per 10 µg/m^3^ for individuals 20 years and older. The RR was not adjusted for the impact of NO_2_ and as such, we estimated the burden from both pollutants separately and emphasize that these should not be added up [41].

### 2.5. Counterfactual Scenario

We estimated premature deaths attributable to air pollution for three counterfactual scenarios:

1. Zero-exposure of the population to air pollution,
2. Air pollution concentrations complying with the WHO air quality guideline values, where in exceedance.
3. Air pollution concentrations complying with the the US NAAQS, where in exceedance.

In the first scenario, the current concentrations, as estimated from the air pollution models, were compared to zero concentrations to demonstrate the overall BoD of ambient air pollution in the city. Note that the zero-exposure scenario is not a realistic scenario and is only defined for comparison purposes. In the second scenario, the current concentrations were compared to the WHO air quality guideline values. WHO recommends that NO_2_ does not exceed 40 μg/m^3^ annual mean and PM_2.5_ does not exceed 10 μg/m^3^ annual mean [33]. In the third scenario, we compared the current concenterations with the US NAAQS, established by the US EPA. The NAAQS annual average limits for NO_2_ and PM_2.5_ are 99 [42] and 12 μg/m^3^ [43], respectively. The RR of mortality in association with NO_2_ and PM_2.5_ was rescaled for the difference between the current concentration levels and the counterfactual concentration levels, as shown in Equation (2).

### 2.6. Sensitivity Analysis

Uncertainties are inherited in variables incorporated in BoD assessment studies, mainly arising from the uncertainty in the baseline health data and the selected ERFs, among others. To explore the range of uncertainty from the variables included in our analysis, including the baseline mortality and ERFs, we ran two uncertainty analyses. First, we estimated the most conservative and most extreme BoD scenarios using the combinations of the lower and upper 95% CI for each of the variables above (baseline mortality and ERFs). These two scenarios are reported in parentheses after the central estimated values of premature deaths attributable to air pollution. Second, we examined the impacts of uncertainty in input data for each variable on premature death estimations. To this end, we reran the analysis for upper and lower 95% CI of each variable, individually, and reported the estimated premature deaths.

## 3. Results

### 3.1. Premature Deaths Attributable to Air Pollution

Table 2 summarizes the estimated premature deaths attributable to NO_2_ and PM_2.5_ in the city of Houston, in 2010. A total of 631 premature deaths for the age group between 30 and 78 years old were attributable to PM_2.5_. Considering the most conservative and the most extreme BoD scenarios, the number of premature deaths attributable to PM_2.5_ varied from 366 to 809 deaths. Similarly, 159 (95% CI: 0–609) premature deaths were attributable to NO_2_. Exceeding the WHO air quality guideline values resulted in 82 (95% CI: 42–95) preventable premature deaths attributable to PM_2.5_. Additionally, 8 (95% CI: 6–10) premature deaths may be attributable to PM_2.5_ exceeding the NAAQS.

**Figure 3:**
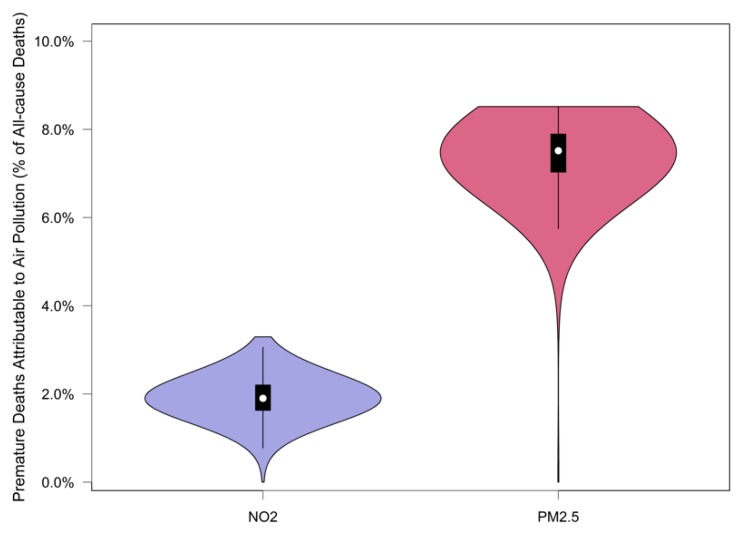
Range and distribution of estimated premature deaths attributable to PM_2.5_ and NO_2_ as a percentage from all-cause deaths for zero-exposure scneario.

**Figure 4:**
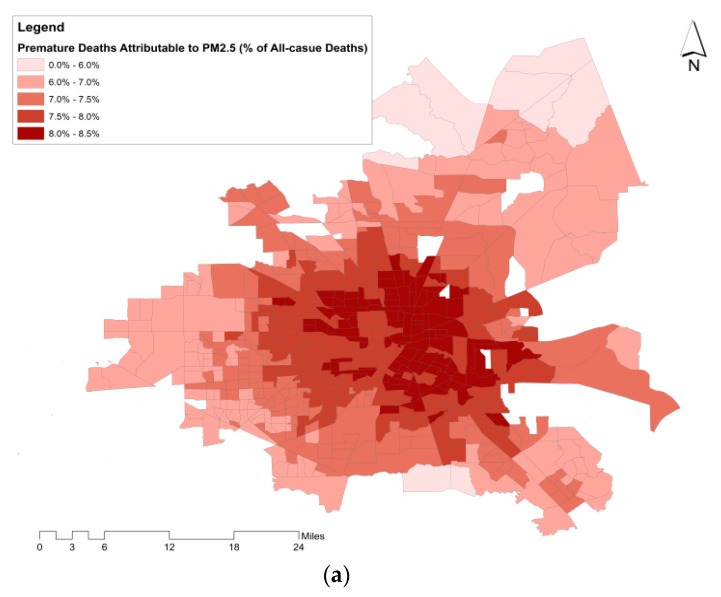

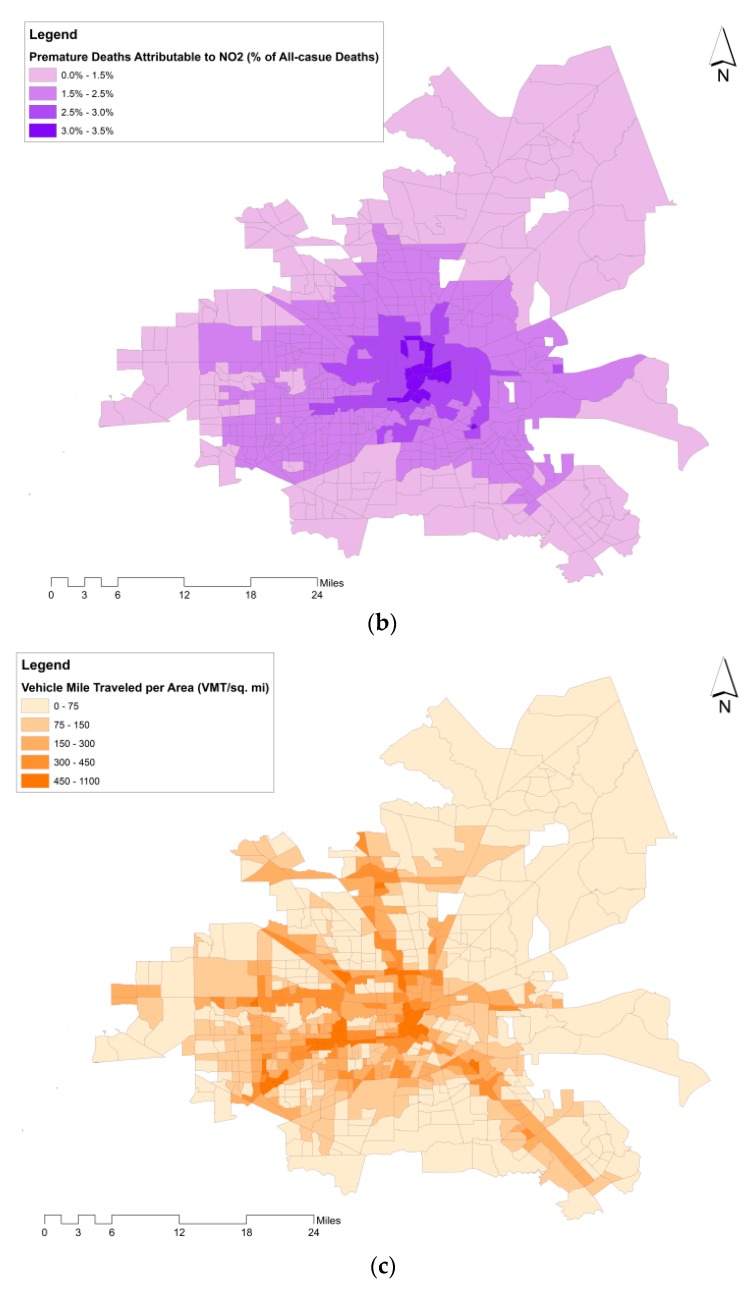

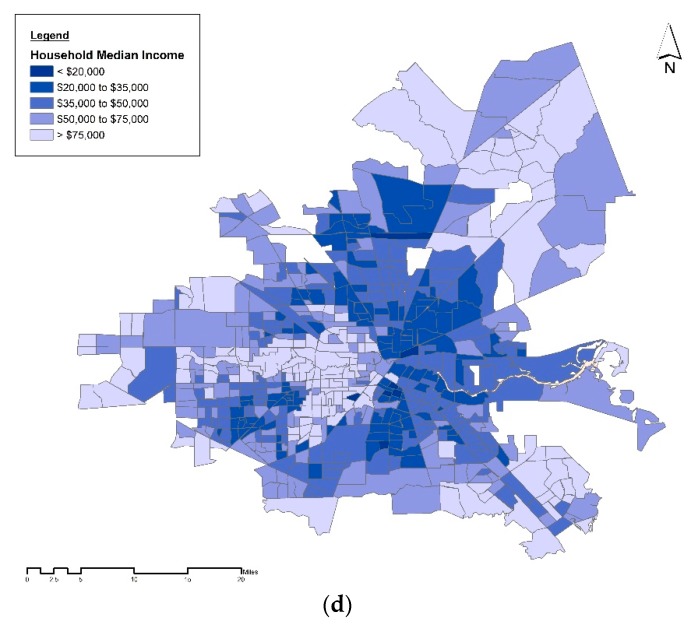
Spatial variation of (**a**) percentage of premature deaths attributable to PM_2.5_ from all-cause deaths, (**b**) percentage of premature deaths attributable to NO_2_ from all-cause deaths across the city of Houston at the census tract level, in 2010. Spatial variation of (**c**) vehicle mile traveled per area, and (**d**) median household income across the city of Houston at the census tract level, in 2010.

Figure 3 illustrates the range of the percentage of premature deaths attributable to air pollution (zero-exposure scenario) from all-cause deaths and its distribution across census tracts. The spatial distribution of premature deaths attributable to PM_2.5_ and NO_2_ (zero-exposure scenario) across census tracts is shown in Figure 4a,b, in the form of a percentage from all-cause premature deaths. The percentage of premature deaths attributable to NO_2_ was higher in census tracts located in the central business district (CBD). Additionally, a spatial similarity is observed between the distribution of air pollutants’ health impacts and VMTA (Figure 4c). 

### 3.2. Premature Deaths Attributable to Air Pollution by Household Income and Road Traffic

**Figure 5:**
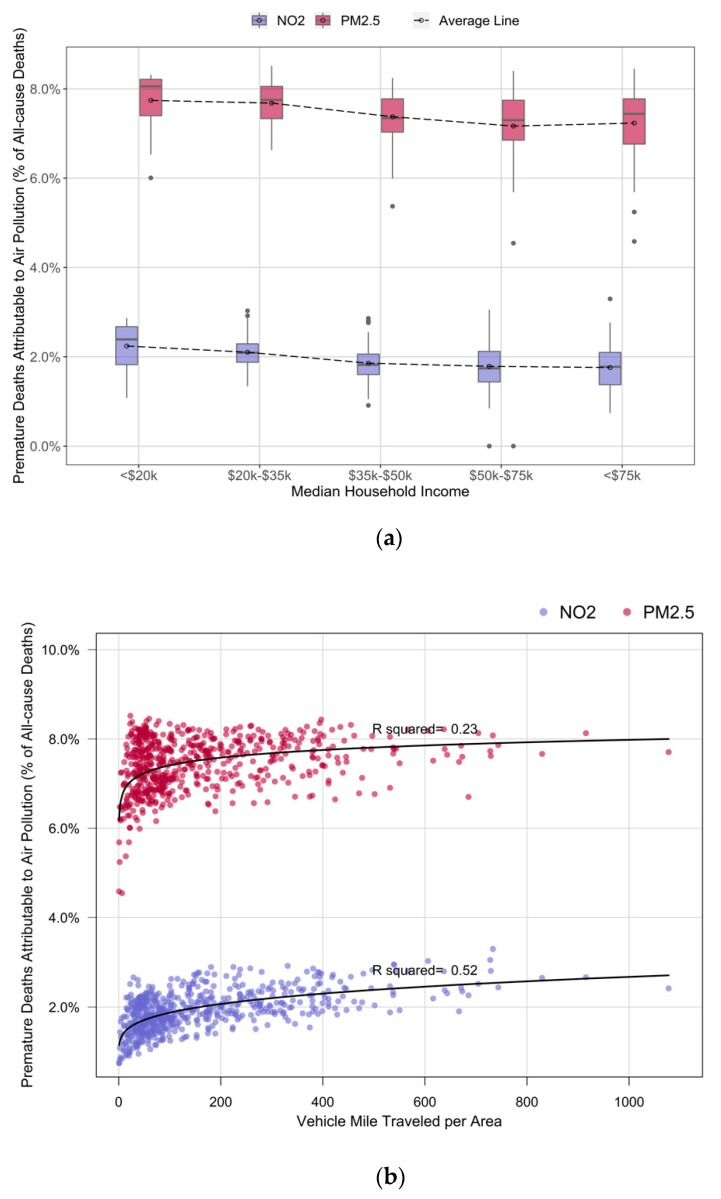
Variation of the percentage of premature deaths attributable to air pollution from all-cause deaths by (**a**) median household income, and (**b**) road traffic.

Figure 4d shows the spatial variation of median household income across the city. The relation between median household income at each census tract and premature deaths attributable to air pollution was further explored. The comparison showed an inverse correlation between median household income at the census tract level and the ratio of premature deaths attributable to air pollution from all-cause premature deaths (the average lines in Figure 5a). In other words, it is expected that the ratio of premature deaths attributable to PM_2.5_ and NO_2_ reduces (by 10% of the estimated ratio (Figure 5a)) with an increase in household income from $20,000 to $75,000.

According to Figure 3, the ratio of premature deaths attributable to PM_2.5_ and NO_2_ at the census tract level can vary from 0.0% to 3.3% and 0.0% to 8.6%, respectively. After a closer look at the spatial distribution of the ratio of premature deaths across the city, a relationship between VMTA and the ratio of premature deaths attributable to air pollutants was shown (illustrated in Figure 5b). This relationship is consistent with the similarity between the spatial variation of the premature deaths attributable to air pollutants and VMTA indicated in Figure 4. The relation between the ratio of premature deaths and VMTA is stronger for the deaths estimated due to NO_2_ compared to PM_2.5_ (R-squared of the best-fitted curve for NO_2_ is 0.52 versus 0.23 for PM_2.5_).

### 3.3. Sensitivity Analysis

The most conservative estimation of premature deaths attributable to NO_2_ resulted in zero deaths. The most extreme estimation resulted in 609 deaths (Table 2). The most conservative and most extreme estimations for premature death attributable to PM_2.5_ resulted in 366 and 809 deaths.

**Figure 6:**
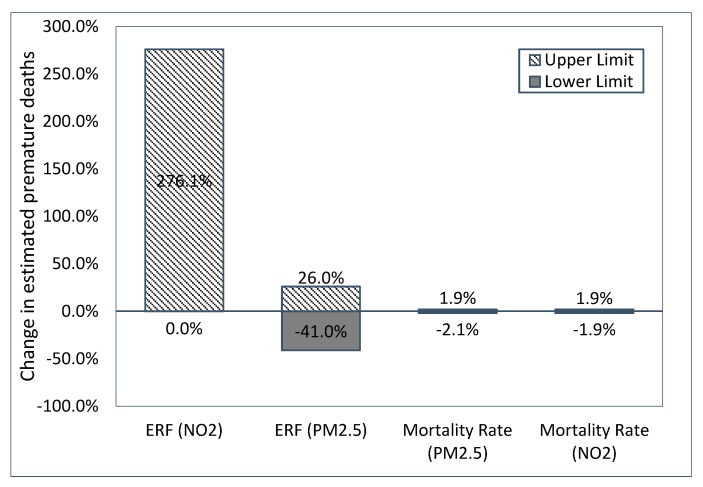
Uncertainty analysis results.

Results of the uncertainty analysis of lower and upper 95th CI of each variable is depicted in Figure 6. The 95% CI of ERFs had the largest role in the uncertainty of estimated deaths, where the estimated attributable premature deaths due to NO_2_ and PM_2.5_ could be changed by up to 276.1% and 41.0%, respectively. The uncertainties in the mortality rates resulted in up to 2.1% deviation in the estimated attributable premature deaths. Overall, more uncertainty was associated with premature death attributable to NO_2_ compared to PM_2.5_.

## 4. Discussion

### 4.1. Key Findings

This study sheds light on the health burden attributable to air pollution in the city of Houston, Texas, in the form of premature deaths. The results showed that, in 2010, 631 (95% CI: 366–809) premature deaths were attributable to PM_2.5_ and 159 (95% CI: 0–609) premature deaths were attributable to NO_2_. The estimated number of premature deaths from PM_2.5_ and NO_2_ can be translated into 7.3% and 1.8% from all-cause premature deaths in the city, respectively. The ratio of premature deaths attributable to PM_2.5_ is higher than the death rate caused by diabetes mellites, Alzheimer’s disease, or motor vehicle crashes in the US (2.8%, 3.4%, and 4.9%, respectively, in 2010 according to [31]), while the ratio of premature deaths form NO_2_ is comparable with the death rate from suicide as well as influenza and pneumonia (1.6% and 2.0% in 2010 according to [31]). Complying with the WHO air quality guideline values for PM_2.5_ (10 μg/m^3^) and the NAAQS criteria for PM_2.5_ (12 μg/m^3^) could prevent 82 (95% CI: 42–95) and 8 (95% CI: 6–10) premature deaths in Houston, in 2010.

We found that the burden of premature deaths from air pollution was higher in the census tracts located in the CBD. Additionally, a similarity between the road traffic spatial variation and the ratio of premature deaths attributable to air pollutants was demonstrated. We showed an inverse correlation between the median household income and the ratio of premature deaths attributable to air pollution. The ratio of premature deaths attributable to air pollution decreases by 10% when the household’s median income increases from $20,000 to $75,000. This is due the higher exposure to air pollutants at census tracts with lower median household income. In addition, the baseline rates of mortality were shown to be higher in communities with lower socioeconomic characteristics [44,45,46]. Unfortunately, we had no other source of mortality data with a finer spatial resolution, which is a common limitation in similar burden of disease assessment studies. Additionally, a positive relation between road traffic and premature deaths attributable to air pollution was shown: the more vehicles passing through a squared mile of a census tract, the higher the risk of deaths from air pollution. The stronger relationship between the ratio of premature deaths attributable to NO_2_ and VMTA compared to the ratio of premature deaths attributable to PM_2.5_ and VMTA is in agreement with the fact that road traffic is responsible for a more significant portion of NO_2_ than PM_2.5_ (68% of NO_2_ in Beijing, China [47] and 40% of NO_2_ in Jamshedpur, India [48] and 56% of NO_2_ in European cities [49] while in the US, traffic contributed to 38% of all nitrogen oxide (NOx) emissions [50]). A higher level of uncertainty was observed in the NO_2_ BoD analysis which is in line with the less precise association between premature deaths and NO_2_ in epidemiological studies. This implies that the evidence on premature mortality attributable to PM_2.5_ is more reliable compared to NO_2_, given the stronger association between PM_2.5_ and mortality, and also importantly the stronger case for biological plausibility.

### 4.2. Strengths and Limitations

In this study, we estimated the premature deaths attributable to air pollutants at the census tract level which enabled us to better investigate the spatial distribution of attributable deaths. The air pollution concentrations were estimated at a relatively high resolution (census block) and then converted to a lower resolution (census tract). We also investigated premature deaths attributable to NO_2_ and PM_2.5_ by the level of road traffic (VMTA) passing through the census tracts and in the catchment area around the census tract where we expect traffic to be most influential. This approach can be considered as a more feasible alternative to full-chain modeling which allows representing the role of traffic-related air pollution in public health. We compared the reliability of the premature death estimates attributable to NO_2_ and PM_2.5_.

However, this study has limitations. We assumed the air pollution concentrations do not spatially vary within a census tract which implies that all populations living in a census tract are exposed to the same average concentration level. To assign the mortality data (available at the county level) to a census tract, we assumed that the mortality rate is constant across the census tract located within a county. Based on that, the mortality cases were distributed between the census tracts according to their population. The extracted ERFs were estimated for adults older than 30 years, and so the potential deaths in the younger population were not quantified. Our approach may, therefore, result in underestimating the health impacts of air pollution in Houston. It is also important to note that the health impacts of PM_2.5_ and NO_2_ cannot be added up because of the overlap in their biological pathways to adverse health outcomes. Although we showed a relationship between road traffic and deaths attributable to air pollution, no conclusion can be drawn on the contribution of traffic-related air pollution to public health impacts as we did not use an approach that allows source apportionment. In addition, while the LUR predicts air pollution with fairly high accuracy, it considers all sources of air pollution and we could not parse out the exact contribution of traffic from other sources in the exposure and associated BoD. Our study used the median household income at the census tract level to stratify the BoD estimates. This socioeconomic indicator can be a proxy for different factors which not only affect the exposure to air pollution but also the human response to those exposures. These factors include ethnicity, diet, stress and violence exposures, and access to health care where ethnicity is particularly important in US [51]. Previous work using the same air pollution models we employed in our analysis showed that ethnicity is indeed an important factor explaining the disparities in NO_2_ concentrations across the contiguous US, which were larger by race/ethnicity than by income [52]. Finally, because of the limitations in the availability of ADT data, 2011 data was used and we assumed that the spatial variation of traffic did not change from 2010 to 2011.

### 4.3. Policy and Research Recommendations

A number of strategies have been discussed in the literature to improve air quality and consequently reduce adverse health impacts, e.g., imposing regulations for air quality control, reducing road traffic-related emissions, and controlling energy generation related emissions as well as greenhouse gas emissions [53]. Among others, traffic-related emissions have been shown to have the most significant impacts on air quality and subsequent health impacts [53]. Therefore, travel demand management (TDM) policies to control the traffic passing through air pollution hotspots in cities, i.e., census tracts with a higher level of exposure and detrimental health impacts, can be efficient solutions to improve public health. Improving public transport, improving infrastructure for active transportation, parking control, road pricing, and prohibiting car traffic are some of the TDM policies that have been practiced in cities [54]. The results of this study underscore the necessity of conducting BoD and health impact assessments of transportation-related projects and designs. The results of such studies can contribute to the cost-benefit analysis of the transportation project and help the city to make more informed decisions.

To estimate the contribution of the traffic-related air pollution more accurately, future research can conduct a full-chain BoD assessment comprising transportation modeling, emission modeling, dispersion modeling, exposure assignment, and finally assessing attributable health impacts. Similar studies can be conducted to evaluate the air pollution and health impacts of new technologies (e.g., electric and hybrid vehicles with less emission rates). Additionally, the comparison between air pollution health impacts and other health risk factors in cities can promote dialogue about air pollution health impacts and support decisions to sustainabily develop cities.

## 5. Conclusions

Quantifying the health impacts of air pollution may support decision-makers, city planners, and public health practitioners to mitigate adverse health impacts and ensure that the most vulnerable populations are not the most impacted. In this study, we quantified the health impacts of air pollution in the form of premature deaths, at the census tract level, in the city of Houston, Texas. We investigated the role of road traffic and socioeconomic status in the health burden attributable to ambient air pollution. A total of 7.3% and 1.8% of all-cause deaths in Houston were attributable to PM_2.5_ and NO_2_, in 2010, respectively. We showed that 0.9% of the premature deaths attributable to PM_2.5_ can be prevented by complying with the WHO air quality guideline value. Deaths attributable to air pollution were higher in the city’s CBD, and in a census tract with a higher level of exposure to road traffic, especially in the case of NO_2_. Additionally, we found that premature deaths attributable to air pollution were higher in areas with lower income households. The findings of this study underline the importance of assessing the BoD attributable to air pollution and its spatial distribution across cities to support sustainable development.

## Figures and Tables

**Table 1:** Summary statistics of input data.

Variable	Sample Size (Census Tracts)	Min	Median	Mean	Max
All-cause premature deaths (persons)	592	1	14	15	61
NO_2_ concentration (μg/m^3^)	592	7.47	19.38	19.52	34.09
PM_2.5_ concentration (μg/m^3^)	592	6.80	11.61	11.41	13.30
VMTA (veh.mi/mi^2^)	592	0.72	99.27	163.04	1077.54
Median household income (dollar)	592	9926	43,352	52,857	214,861

**Table 2:** Premature deaths attributable to air pollution in Houston.

Counterfactual Scenario	Premature Deaths Cases (95% CI)	Air Pollutant	Counterfactual Concentration (μg/m^3^)	Adjusted RR Associated with 10 μg/m^3^ Increase (95% CI)	Attributable Premature Deaths (95% CI)	% of Attributable Premature Deaths to All-Cause Deaths (95% CI)
Zero-exposure scenario	8667 (8499–8834)	PM_2.5_	0	1.07 (1.02–1.12)	631 (366–809)	7.3% (4.3%–9.2%)
		NO_2_	0	1.01 (0.99–1.04)	159 (0–609)	1.8% (0.0%–6.9%)
Complying with WHO guidelines	8667 (8499–8834)	PM_2.5_	10	1.07 (1.02–1.12)	82 (42–95)	0.9% (0.5%–1.1%)
		NO_2_	40	1.01 (0.99–1.04)	0 ^1^ (0–0)	0.0% (0.0%–0.0%)
Complying with NAAQS	8667 (8499–8834)	PM_2.5_	12	1.07 (1.02–1.12)	8 (6–10)	0.1% (0.0%–0.1%)
		NO_2_	99	1.01 (0.99–1.04)	0 ^1^ (0–0)	0.0% (0.0%–0.0%)

^1^ NO_2_ concentration across the city was lower than the WHO air quality guideline values.
